# *Listeria monocytogenes* in Irrigation Water: An Assessment of Outbreaks, Sources, Prevalence, and Persistence

**DOI:** 10.3390/microorganisms10071319

**Published:** 2022-06-30

**Authors:** Samantha Gartley, Brienna Anderson-Coughlin, Manan Sharma, Kalmia E. Kniel

**Affiliations:** 1 Department of Animal and Food Science, University of Delaware, Newark, DE 19716, USA; sgartley@udel.edu (S.G.); briennaa@udel.edu (B.A.-C.); 2 Environmental Microbiology and Food Safety Laboratory, Beltsville Agricultural Research Center, Northeast Area, Agricultural Research Service, US Department of Agriculture, Beltsville, MD 20705, USA; manan.sharma@usda.gov

**Keywords:** foodborne illness, bacteria, contamination, environment

## Abstract

As more fresh fruits and vegetables are needed to meet the demands of a growing population, growers may need to start depending on more varied sources of water, including environmental, recycled, and reclaimed waters. Some of these sources might be susceptible to contamination with microbial pathogens, such as *Listeria monocytogenes*. Surveys have found this pathogen in water, soil, vegetation, and farm animal feces around the world. The frequency at which this pathogen is present in water sources is dependent on multiple factors, including the season, surrounding land use, presence of animals, and physicochemical water parameters. Understanding the survival duration of *L. monocytogenes* in specific water sources is important, but studies are limited concerning this environment and the impact of these highly variable factors. Understanding the pathogen’s ability to remain infectious is key to understanding how *L. monocytogenes* impacts produce outbreaks and, ultimately, consumers’ health.

## 1. Introduction

There has been a rise in recalls and outbreaks related to fresh produce, with the percentage of illness attributed to produce being as high as 46% [1,2,3,4,5,6,7]. Several studies have examined the presence of *Salmonella* or shigatoxigenic *E. coli* in the environment, associated with fresh produce [8,9,10,11,12], whereas fewer studies have addressed the prevalence and origins of *L. monocytogenes* contamination. Though it is believed that *Listeria monocytogenes* survives in biofilms, and that these play an important role in the contamination in packing houses and processing facilities [13], this is not the only pathway by which fresh produce may come in contact with the pathogen [14,15,16]. Irrigation water is a route by which fresh produce may become contaminated, as it often makes direct contact with the edible portion of the plant during application.

The presence of bacterial pathogens, such as *L. monocytogenes*, in irrigation water is already a pressing issue for producers and consumers of raw agricultural commodities around the world [8,17,18,19,20,21]. Several studies have shown that irrigation water sources can contain *L. monocytogenes* [22,23,24]. As the population of the United States continues to grow, the need for more fresh fruits and vegetables does too. This means a larger draw on the limited sources of water, for irrigation and processing, that growers have access to. Water resources are already strained in several parts of the country, and, with an increasingly variable climate, areas such as the Mid-Atlantic region might also experience the need to use more diverse water sources for irrigation [1,2,3,4,5]. With the use of these additional water sources, including lakes, ponds, rivers, streams, lagoons, recycled, and reclaimed waters, microbiological quality becomes a concern.

A nationwide genomic analysis of *Listeria* focused on microbial population ecology and pangenome evolution, suggesting core genes across the genus may be important drivers of adaptation [25]. It is likely that more comprehensive genomic analyses will continue to study the role of environment and bacterial persistence in relation to outbreaks and cases of illness. However, not much is known about the survival of *L. monocytogenes* in water sources and the potential impacts of the variable gene expression, including the internalin A (*inlA*) gene.

This review focuses on the occurrence of *L. monocytogenes* in a variety of water sources, the potential role of irrigation water and the farm environment in outbreaks related to produce contaminated with *L. monocytogenes*, and the presence of genetic sequence differences within isolates obtained from water, food commodities, and food processing facilities.

## 2. *L. monocytogenes*—Foodborne Pathogen and Produce Outbreaks

*Listeria monocytogenes* is a gram positive, non-spore-forming firmicute bacterium that grows at temperatures from 0–45 °C, is able to attach to surfaces, and has the ability to cause invasive infections [26,27]. Although disease caused by this organism only has an incident rate, on average, of 0.3 per 100,000 people, of the over 2300 cases annually, 92% require hospitalization, and 16% end in death [27,28]. The risk of dying from listeriosis is highest for individuals with compromised immune systems, young children, the elderly, and pregnant women. Depending on who and where in the person the infection occurs, symptoms can present in a variety of ways. Similar to other foodborne pathogens, it can present with a fever and diarrhea, but this type of infection is rarely diagnosed [27]. More common is the invasive listeriosis in which the bacteria have passed beyond the gut. This can also have varying symptoms depending on if the individual is pregnant. Pregnant women may experience flu-like symptoms, e.g., muscle aches, fatigue, and a fever, but this can lead to serious results including still-birth, miscarriage, premature delivery, or life-threatening infection of the newborn. Other individuals with invasive listeriosis may present with symptoms such as headache, stiff neck, confusion, loss of balance, and convulsions that may be in addition to fever and muscle aches. Symptoms, on average, may take up to 1–4 weeks before presenting [27]. These symptoms, however, can be misinterpreted as other illnesses and can take long periods of time to present, so misdiagnosis can occur and lead to outbreaks not being identified. *L. monocytogenes* has been historically linked to deli meats, cheeses, and hot dogs [27], but it has been linked to produce, including cantaloupes, sprouts, frozen vegetables, and broccoli, in more recent outbreaks [6]. Actual or potential *L. monocytogenes* contamination has resulted in numerous recalls of fruit and vegetable crops, including stone fruits [29] and leafy greens [30]. *Listeria* spp. are ubiquitous in the environment, and frequently, they can be isolated from soil, water, decaying vegetation, and animal sources [10,11,12,19,20,23,24,31,32,33,34,35,36,37,38,39,40,41,42,43,44,45,46,47,48,49,50]. Thus, it is not surprising that *L. monocytogenes* can be detected in the farm environment and, ultimately, contaminate raw agricultural commodities [51].

In the past, cases may have been considered sporadic or unrelated. More recently, improvements in epidemiological information, coupled with increased microbial testing and greater awareness, have made it easier to identify connections between cases and follow them back to a common source [52,53,54]. Applying these tools, retrospectively, to previous outbreaks, where possible, may help shorten or prevent future outbreaks.

Outbreaks associated with produce contaminated with *L. monocytogenes* have occurred several times in recent years. One occurred in the summer to autumn months of 2011. This multistate outbreak included 147 people, from 28 states, infected with any of five outbreak-associated strains, and it was traced back to whole and fresh cut cantaloupes originating from Jensen Farms’ packing facility in Colorado, where the *L. monocytogenes* isolates were found, in samples from both equipment and cantaloupe [55,56,57].

Three years later, in the summer of 2014, five people from two states were hospitalized with listeriosis. Two of the five people interviewed had eaten bean sprouts in the recent past, and ultimately, two individuals died. Whole genome sequencing showed that *L. monocytogenes* strains, isolated from environmental swabs taken during inspections at the Wholesome Soy Products, Inc., were highly similar to clinical strains from the five people hospitalized. Testing, as part of routine inspections of the company’s facility by the FDA, revealed that mung bean sprouts and sprout irrigation water were contaminated with *L. monocytogenes*. Twenty-five environmental swabs also tested positive for this organism. Numerous unsanitary conditions and poor equipment maintenance were cited, causing the company to issue a recall. Ultimately, the company was forced to cease production, and it closed by November of 2014 [58].

Caramel apples were the next produce vehicle to cause a multistate listeriosis outbreak later that same fall (2014). Thirty-five cases were reported, with 34 individuals hospitalized and seven deaths. Pulsed-field gel electrophoresis (PFGE) was used to determine there were two outbreak clusters, and whole genome sequencing determined that there were two main isolates: one within each cluster. It was determined that all cases were related, as one individual had both strains. Twenty-eight people (90%) reported eating pre-packaged caramel apples. In January, Bidart Bros. recalled granny smith and gala apples because environmental testing in their apple-packing facility indicated contamination with *L. monocytogenes.* The FDA confirmed, via pulsed-field gel electrophoresis and whole genome sequencing, that these strains were the same as the outbreak strains. These strains also matched those from apples tested along the supply chain [59].

In 2016, pre-packaged salads led to an outbreak of listeriosis that occurred over nine states with nineteen cases, where all nineteen individuals were hospitalized, and one person died. Whole genome sequencing revealed that all the clinical isolates were closely related. The Public Health Agency of Canada also reported people infected by the same outbreak strain of *L. monocytogenes*. The Ohio Department of Agriculture collected packaged salads from retail locations as a part of their routine product sampling program and isolated *L. monocytogenes*, which were genetically similar to the outbreak strains. Finally, it was concluded that pre-packaged salads at Dole’s processing facility in Springfield, Ohio was the likely source of the outbreak, and Dole issued a recall of the salad mixes processed at the facility [30].

In 2016, frozen vegetables were identified as cause of a multistate outbreak across four states with nine cases. Three cases were identified in 2016, but the other six were determined by retrospective review of the PulseNet database for similar strains and potential food vehicles. All nine cases were hospitalized, and ultimately, three of the persons died. Evidence based on questionnaires of foods that were consumed linked back to frozen vegetables produced by CRF Frozen Foods. The Ohio Department of Agriculture isolated *L. monocytogenes* from frozen corn, as well as frozen peas, and it was revealed that these isolates were identical to clinical isolates from two of the nine cases. In addition, *L. monocytogenes* isolates linked to outbreak cases were found by the FDA during environmental sampling of the Oregon Potato Company. The Oregon Potato Company thus recalled its wholesale onion products. From the CFR Frozen Foods recall, a total of 350 consumer products from 42 brands and an additional 100 other products that used the frozen vegetables were recalled [60].

In 2020, enoki mushrooms from H&C Food Inc., Guan’s Mushroom Co., and Sun Hong Foods Inc., in the Republic of Korea were recalled due to a *L. monocytogenes* contamination, which caused 31 hospitalizations and four deaths in the 36 reported cases across 17 states [61]. Recently (February 2022), enoki mushrooms have been recalled by the Washington State Department of Health for potential *L. monocytogenes* contamination after routine testing, performed by the Michigan Department of Agriculture and Rural Development, resulted in detection of the pathogen [62].

In 2021, two separate *L. monocytogenes* outbreaks were investigated by the CDC, including Dole brands and Fresh Express brand packaged salads. In the Dole outbreak, 17 infections, with 13 hospitalizations and two deaths across 13 states were identified, while the Fresh Express outbreak resulted in 10 infections, all of which were hospitalized, with one death across eight states. The Dole outbreak was linked back to a contamination of harvesting equipment used for iceberg lettuce and other produce [63,64]. *L. monocytogenes* contaminations causing the outbreaks and recalls were traced back to steps in the processing or packing facilities. However, the route of *L. monocytogenes* contamination into the facility or processing step was not identified. It has been speculated that sources could have included produce from the field, not properly treated wash water, movement of workers, or pests [65]. Traceback can be difficult due to issues, ranging from patient interviews being limited or incomplete, samples not having been collected from patients, and the time it takes to culture the pathogen, to the fact that different typing methods (e.g., whole genome sequencing or PFGE) may provide different degrees of specificity about isolates [30,66]. As traceback can take weeks or longer, products or environmental conditions may have changed or been lost, creating difficulty in identifying the source of the contamination [67].

Currently (June 2022), there is an ongoing investigation, recall, and potentially contaminated ready-to-eat salads from Northern Tier Bakery, LLC, located in Minnesota, United States. Two salad products were recalled after the company’s routine testing resulted in *L. monocytogenes* detection, and the USDA’s Food Safety Inspection Service was notified [68].

Although no *L. monocytogenes* outbreaks have been attributed to irrigation water, there have been instances where irrigation water and the associated sediment have harbored the outbreak isolates, suggesting the potential source and cause of these outbreak. This supports the possibility for irrigation water to lead to the contamination of produce. In one such instance in 2018, with a Romaine lettuce outbreak in Yuma, AZ, almost two months passed between the onset of the outbreak and when environmental sampling took place, allowing for possible connections to be lost. Isolates of *Escherichia coli* O157:H7, associated with contaminated Romaine lettuce, were identified in an irrigation canal that connected multiple farms in Yuma, Arizona [67,69,70]. Later that year, Romaine lettuce was, again, connected to *Escherichia coli* O157:H7 in the sediment of an irrigation source used in California [71].

The presence of bacterial pathogens in fresh produce items remains an important issue, and the likelihood of *L. monocytogenes* contamination remains largely unknown. A sampling of commercially sold vegetables (*n* = 306) in Malaysia found that *Listeria* spp. were detected in 33.3% of vegetables, with 22.5% of vegetables containing *L. monocytogenes,* specifically [72]. Recently, Consumer Reports conducted a survey of the presence of *L. monocytogenes* in leafy greens sold at major supermarkets [73]. This study consisted of 284 samples of fresh greens, such as lettuce, spinach, and kale that were either bagged, un-bagged, branded, unbranded, or pre-washed, collected at retail. Among the 284 samples tested, six were positive for *L. monocytogenes*. Two of the six positive samples originated from products that were labeled as “triple-washed”, while the rest were from un-bagged products. Half of the samples were labeled under a brand name, while the other three samples were unbranded. One sample had an isolate that was genetically linked to at least two cases of listeriosis that had been reported to the Center for Disease Control and Prevention, although it was not known whether those cases were associated with the consumption of leafy greens [73]. Articles such as the one in Consumer Reports, and subsequent news outlets that pick them up, remind the public of the risks associated with produce and are an incentive for the food industry to continue to assess the potential origins of contamination by *L. monocytogenes* and the means of prevention.

## 3. Presence and Prevalence of *L. monocytogenes* in Water and Natural Environments

As the need for additional irrigation water sources grows, multiple surveys have evaluated watersheds for the presence and prevalence of *Listeria* spp. around the globe, such as the central coast of California [10], New York State [11,12,20], the Mid-Atlantic, USA [74], Canada [24,42], Germany [37], Austria [38], South Africa [8], Demark [75], and India [47]. Across these varying environments, the occurrence of *L. monocytogenes* ranged from 0% to 67%, with sample sizes ranging from 26 to 1405 water samples included in the studies. The authors of these studies attributed the frequency at which the pathogen was present to factors such as temperature, season, and water parameters, e.g., chemical oxygen demand, surrounding ecosystems, including urbanization, agricultural animals and their contaminated manures, and the degree of human activity [8,10,11,12,20,24,37,38,41,47,75]. Location, quantity and volume of samples, and the factors attributed that are associated with *L. monocytogenes* prevalence, from these studies, are described in Table 1. These factors may influence the amount of stress experienced by the *Listeria* cells, thus influencing their ability to survive and move through the different parts of the food chain [76]. Although multiple studies of *L. monocytogenes* in water environments have been published [8,10,11,12,20,24,37,38,42,47,75], only three of them quantified levels in the water samples collected. Lang-Halter et al. (2016) [37], Sharma et al. (2020) [74], and Achemafour et al. (2021) [77] reported low levels in surface water creeks, ponds, tidal brackish river, four non-tidal fresh rivers, and a tidal fresh river, with MPN values ranging from <0.3–>110 MPN/g and <0.03–>11 MPN/100 mL, respectively. Studies that provide an indication of the level of *L. monocytogenes* can be used to evaluate potential risks associated with irrigation water and irrigated products.

*L. monocytogenes* is found in a variety of water sources around the world. However, few studies have reported the survival of *L. monocytogenes* in water over time. *L. monocytogenes* has been shown to survive for at least 40 days at 20 °C and over 100 days at 4 °C in river water downstream from a meat industry plant, respectively [80]. Similar results were seen in Danish freshwater and seawater, with better survival at 5 °C than at 20 °C for all three strains observed [75]. Additionally, it was found that the three *L. monocytogenes* strains decrease to undetectable levels after 26 days (two strains) in the 20 °C seawater and 40 days (one strain) in autoclaved and filtered (0.2 µm) seawater. No significant differences in the persistence were observed between natural freshwater and autoclaved and filtered freshwater. In comparison, artificial water sources (Instant Ocean [IO] and Phosphate Buffered Saline [PBS]) were inoculated with the three *L. monocytogenes* strains, and their levels decreased in the IO over time but were still detectable throughout the entire study, while the *L. monocytogenes* levels remained constant over time in the PBS. The differences between the reductions in natural versus autoclaved or filtered versus artificial water were attributed to microbial competition and protozoan grazing [75]. These results show that, depending on the type of water used in production, it can influence the survival and persistence of *L. monocytogenes* further down the food chain.

Most of the studies listed in Table 1, in addition to the collection of water samples, also screened soil samples in adjacent fields. *L. monocytogenes* has been detected in 11% (30/263), 13% (5/40), 6% (28/467), and 9% (16/178) of soil samples, respectively, compared to 30% (22/74), 30% (10/33), 0% (0/68), and 28% (48/174) of water samples taken concurrently, respectively [12,17,18,38]. More frequent recovery of *L. monocytogenes* from water may have occurred because of the ability to analyze larger volume samples of water compared to soil, as well as less microbial competition in water samples compared to soil samples for *L. monocytogenes.* It may also be due to inherent biotic or abiotic factors differing between soil and water, which may allow for *L. monocytogenes* to survive and thrive better in water than in soil. Nevertheless, the parameters that were found to be important in soil (e.g., temperature, microbial population, strain mobility) are likely to be important to survival in a water environment [81].

## 4. *Listeria monocytogenes* and Overall Pathogen Presence: Reclaimed and Recycled Waters

In addition to surface water sources for irrigation, recycled and reclaimed water sources are also of great interest to meet the ever-growing water demands of agriculture. Concerns with recycled wastewater include the potential to carry foodborne pathogens if the water is not treated properly or the treatment process is not validated. Surveys of *Listeria* spp., specifically *L. monocytogenes,* in wastewater effluent from treatment plants around the world have shown a range of levels in these waters throughout the plants’ multi-step treatment processes [82,83,84]. In a study out of South Africa, researchers found that, among positive wastewater effluent samples, *Listeria* spp. concentrations varied from 2.9 × 10^0^ to 1.2 × 10^5^ CFU/mL, and unlike surface water samples, they were not significantly correlated with season [50,84]. A study in Spain found that lettuce harvested from wastewater-irrigated plots showed significantly higher levels of foodborne pathogens, including *L. monocytogenes*, compared to plants irrigated with a groundwater source that provides drinking water [17]. However, a study conducted in Italy, which compared ground water and secondary treated wastewater, found that the reuse of food industry wastewater, for the irrigation of agricultural crops, did not post an increased risk of microbial contamination to fruits and vegetables. The secondary treated wastewater consisted of the company’s sewage and industrial vegetable processing water, collected from a secondary settler but prior to the chlorine treatment, at the on-site wastewater treatment plant. The crops used in this study were tomatoes and broccoli, and although pathogens were found in the soil and on broccoli during the season, no pathogens were detected at time of harvest on either type of crop [85]. These different outcomes may be due, in part, to the type of treatment that the wastewater had undergone, the crop varieties that were used in the study, such as tomatoes and broccoli, which are normally transplanted to soils, while lettuce is normally started from seed, likely increasing contact with the soil. Once these crops are contaminated, trying to remove or inactivate the total pathogen load by washing can be difficult [86]. Cut Romaine lettuce and whole cantaloupe rinds that were inoculated with *L. innocua* at various levels have shown to have a maximum of 1 log and 0.5 log reduction when washed with chlorinated water and tap water only, respectively. Less reduction was observed from the cantaloupe surface compared to the cut Romaine lettuce for both chlorinated and tap water, which was attributed to the differences in surfaces between the two produce types [22]. Thus, preventing the introduction of bacterial pathogens to produce, through different types of irrigation sources, is important.

Wastewater treatment plants regularly discharge effluent into surface waters [87], which may impact agricultural environments. Current EPA regulatory standards do not include a standard for *L. monocytogenes* in discharged effluent; without these standards, it is unknown how much *L. monocytogenes* could be introduced into surface waters used for irrigation in the United States, as well as globally [87,88].

## 5. Persistence of *Listeria* spp. in Water, Soil, and on Crop Surfaces

Other studies recorded observations regarding the survival and transfer of *L. monocytogenes* and surrogates in a field trial setting. Soil inoculated with *L. innocua,* a commonly employed surrogate for *L. monocytogenes*, supported a 7 log reduction over 90 days. This led to the conclusion that, between the application of untreated soil amendments (animal manure) as a fertilizer, which can contain *L. monocytogenes*, and the harvest of a specific fruit/vegetable, there should be a sufficient amount of time to ensure the reduction in *L. monocytogenes* in soils, assuming that the source of contamination occurred at the beginning of–and only once during–the growing season [51]. Given the length of time of survival, as previously described [80], irrigated crops could be re-contaminated throughout a growing season, either from the water directly or from the application of water to the soil, which could then become a reservoir to continually contaminate the crops. A surveillance study did not find *L. monocytogenes* in soil or manure samples, but found it in one manure-contaminated water sample and in one lettuce sample from a field irrigated with the manure-contaminated water 3, 7, and 21 days before harvesting. The authors speculated that, because of the low incidence of *L. monocytogenes,* links to contamination could not be determined between either the lettuce or soil and the type of irrigation used [89].

Another study looked at the potential risk from water contaminated with high concentrations of *L. innocua*, a surrogate for *L. monocytogenes*. Contaminated irrigation water and manure were evaluated as a vehicle to transfer *L. innocua* on to lettuce leaves and the surrounding soil. *L. innocua* survived in soil for nine weeks at high concentrations (10^5^ CFU/gdw in fall and 10^3^ CFU/gdw in spring). Higher levels of survival were associated with higher levels of temperature and humidity especially in the fall. When lettuce was sprinkled with the contaminated water, *L. innocua* levels were initially high, but under field conditions, they decreased to undetectable levels. In addition, transfer from contaminated soil and irrigation water was observed to the lettuce leaves [90]. These results show that, although *L. innocua* levels were reduced on the leaves over time from the direct application of water, the potential risk of contamination prior to harvest remains, either from the original contaminated water source (if irrigation is applied again) or from the contaminated soil. These findings emphasize the need for water sources of high microbiological quality for growing raw agricultural commodities without the risk of contamination.

Microbial competition plays a role in the survival and proliferation of bacteria in soil, water, and on leafy greens and may affect levels of *L. monocytogenes* in pre-harvest environments. In addition to the points described above, it has been suggested that the reduction on the lettuce leaves, from high levels to undetectable levels over time, at field conditions may be due to drying, solar radiation, and/or microbial competition [90]. In a separate study, water from an irrigation canal and two rivers, used to irrigate cauliflower and broccoli, were evaluated for bacterial pathogens and aerobic colony counts. The authors found that low levels of aerobic colony counts were positively correlated with indicating a high probability of *L. monocytogenes* on the vegetables [8]. These studies also support the findings of McLaughlin et al. (2011): that the presence of background microbiota influences the growth of *L. monocytogenes*. In their study, they added *L. monocytogenes* to a sterile soil and to the same soil to which an aerobic microbial mixture had been added. In the latter, the *L. monocytogenes* population decreased faster than in the sterile soil [81]. This outcome may have been due to competition with, or inhibition of, *L. monocytogenes* by the added microorganisms, which could also occur in a natural water environment.

## 6. Implication of Internalin A in *L. monocytogenes*

Traditional means of bacterial culture give an indication of viability and culturability, but they do not inform about infectivity. In vitro infectivity assays for *L. monocytogenes* include assessment in cell culture or animal models [26,91,92]. *L. monocytogenes* has the ability to cause an invasive infection through a group of proteins called internalins. Internalin A, which is encoded by the *inlA* gene [26], allows *L. monocytogenes* cells to bind to the host cell similarly to the host’s natural cell-to-cell adhesions (E-cadherin). This host cell/bacterial cell adhesion causes the host cell to engulf the bacterial cell by a zipper mechanism, leading to the host cell’s actin cytoskeleton rearrangement and envelopment of the bacteria in a vacuole within the host cell. Once inside, the *L. monocytogenes* cell takes approximately 30 min to escapes the vacuole. This happens by *L. monocytogenes* producing a type of hemolysin called listeriolysin-O (LLO). This pore-forming toxin, also mediated with either a phosphatidylinositol phospholipase C (PlcA) or a phosphatidylcholine phospholipase C (PlcB), depending on if there is a single or double membrane vacuole, helps to degrade the vacuole and release the bacterial cell. Once released into the cytosol, *L. monocytogenes* recruits the host cell’s own actin by using the bacterial cells’ surface protein ActA. This protein uses structural mimicry to host cell’s WASP (Wiskott–Aldrich syndrome protein, a family of proteins that function by activating Arp2/3 an actin nucleating complex and promotes mammalian crosslinking of actin filaments), which is used to recruit and activate the mammalian cells’ actin, which propel the bacterial cell into the adjacent cell, evading the host’s immune system [26]. If Internalin A, which initiates this invasion process, is not functional because the *inlA* gene has deletions and/or premature stop codons, the ability of *L. monocytogenes* to invade a human’s cell is greatly reduced [43]. The occurrence of *L. monocytogenes* strains, with mutations in the *inlA* gene, suggests that such mutants might have an advantage over more virulent cells under certain conditions. This genetic system should be further explored to increase our understanding of the effects in natural water environments, similar to the pan-genome evolution being assessed in soil environments [25].

Multiple studies have looked at the *inlA* gene alleles present in bacterial isolates of various origins, including foods, food processing environments, animal and human clinical samples, and the natural environment [18,93]. Manuel et al. (2015) [18] found that isolates from food and food environmental samples were more likely to contain premature stop codons (PMSC) in the *inlA* gene, unlike human and animal clinical case isolates with fully intact genes. This may be a reason supporting the lack of clinical cases when *L. monocytogenes* has been identified in a food processing facility. Similarly, Nightingale et al. (2005) [43] found that, in the Pathogen Tracker database [94] of over 5000 isolates, the six ribotypes associated with premature stop codons were more commonly associated with foods than with human clinical cases. It has also been found that the isolates from human and food sources, when compared after a plaque assay for plaque area size and infectivity by colony forming units (CFU) per plaque forming units (PFU), are from two unique but overlapping populations. It showed that the clinical human isolates, as a group, had a higher average plaque area, as well as a higher infectivity with a lower CFU per PFU than the food isolates [93].

Within the Central California Coast, five watersheds were evaluated for the presence of *L. monocytogenes* over two years, and the *inlA* gene, sequenced from isolates to determine if these were fully intact *inlA* genes, had a version of a premature stop codon (PMSC) or other deletion in the gene. Among the 112 isolates collected, 90% (101/112) had intact *inlA* genes, only three isolates had a previously known PMSC, and eight isolates had a nine nucleotide deletion that had been previously described in a clinical case [19]. Although the majority seemed to produce fully intact proteins, this remains speculation until these isolates are exposed to cells and infection is determined. Agricultural water and soil samples, collected in Eastern Cape Province, South Africa, yielded 117 and 183 presumptive *L. monocytogenes*, respectively. Through PCR analysis, eight isolates from water and 12 isolates from soil were confirmed to be *L. monocytogenes*, and *inlA* was detected in all isolates, along with eight other virulence genes [95].

Other studies have characterized the potential virulence of a functional internalin A in addition to surveying the presence of *L. monocytogenes* in feces of livestock, wildlife, humans, and surface waters in Ontario, Canada. A representative set of isolates collected from animal and human feces, as well as surface water, were exposed to Caco-2 cells for 24 h to assess infectivity through invasive entry. The number of plaques were compared to the initial bacterial inoculum and expressed as a percentage. In the fecal samples studied, 59% of the representative set of 15 isolates were found to exhibit potential virulence linked to a functional internalin A, which had a higher average percent of entry (9.3%) than isolates with a truncated internalin A (1.3%). In the surface water study, 50% of the 14 representative isolates that were exposed to Caco-2 cells were assayed to possess a functional internalin A. A portion (28%) of the isolates were found to be moderately-to-highly virulent by plaque assay [41,42]. Currently, there are few data on the presence and form of *inlA* in *L. monocytogenes* isolated from water sources in other regions of the United States, as well as from around the world. These critical data gaps should be addressed to obtain a complete understanding of the risk associated with *L. monocytogenes* in environmental waters.

## 7. Conclusions

*Listeria monocytogenes* is an important foodborne pathogen. It has been found in pre-harvest and on-farm environments, as well as linked to outbreaks associated with contaminated produce around the world. Environmental surveys are important ways of increasing our understanding of the presence and prevalence in irrigation water sources and their surrounding environments. Studies should continue assessing globally important watersheds, especially those critical to the irrigation of fruits and vegetable crops. As survival has been studied in soils, food, and processing environments, so too should survival be studied in various water sources to get a more critical understanding of prevention in potentially transmissive ecosystems. Emphasis should be placed on reused water, treated wastewater or processing water, which can enhance current irrigation water supplies. Several questions remain regarding water conditions that may enhance survival, the role of microbial competition, and how virulence factors may support survival and transmission to consumers along the farm-to-fork continuum.

## Figures and Tables

**Table 1:** Presence and factors attributing to *Listeria monocytogenes* in water environments.

Country	Positive/Total Samples (% Positive)	Type of Water	Factors Contributing to Presence	Reference
USA (California)	605/1405 (43)	Lake, stream, river, pond	Point-source, roaming animals, high run-off from heavy rains	[10]
USA (New York)	48/174 (28)	Surface and engineered	Farm, season, sample type, temperature	[12]
USA (New York)	22/74 (30)	Irrigation (engineered, pond, river) and non-irrigation (pond, ditch, river)	Water source (non-irrigation vs. irrigation)	[11]
Canada (Ontario)	32/134 (10)	Surface (river)	Proximity to upstream dairy farm, degree of crop land	[42]
Germany	24/36 (67)	Creek and pond	Area rich in agriculture and plant life	[37]
Austria	0/68 (0)	River and pond	Proximity to agricultural lands, urban environments	[38]
South Africa	19/36 (53)	Irrigation canal and river	High chemical oxygen demand detected	[8]
India (Varanasi)	8/100 (8)	River Water	Proximity to large human population	[47]
Canada (Nova Scotia)	56/329 (17)	Rural and urban watersheds (river and lake)	Rural agricultural watersheds	[24]
USA (New York)	10/33 (30)	Irrigation (well, pond) and non-irrigation (ditch creek)	Sources of water, more likely in non-irrigation sources	[20]
USA (Mid-Atlantic)	53/171 (31)	Pond, non-tidal fresh, tidal fresh, tidal brackish, reclaimed	Water source (environmental vs. reclaimed)	[74,77]
Switzerland	25/191 (13)	River, stream, inland canal	Agricultural area and dense human populations	[78]
Denmark	2/26 (8)	Stream, freshwater fish farm, seawater fish farm, municipal water	Prevalence increased with degree of human activity	[75]
USA (New York)	86/209 (41%)	Pond, stream, and wildlife fecal samples	Small farm with wildlife intrusion	[79]

## Data Availability

Not applicable.
